# Complete genome sequence of *Arthrobacter phenanthrenivorans* type strain (Sphe3)

**DOI:** 10.4056/sigs.1393494

**Published:** 2011-04-29

**Authors:** Aristeidis Kallimanis, Kurt M. LaButti, Alla Lapidus, Alicia Clum, Athanasios Lykidis, Kostantinos Mavromatis, Ioanna Pagani, Konstantinos Liolios, Natalia Ivanova, Lynne Goodwin, Sam Pitluck, Amy Chen, Krishna Palaniappan, Victor Markowitz, Jim Bristow, Athanasios D. Velentzas, Angelos Perisynakis, Christos C Ouzounis, Nikos C. Kyrpides, Anna I. Koukkou, Constantin Drainas

**Affiliations:** 1 Sector of Organic Chemistry and Biochemistry, University of Ioannina, Ioannina, Greece; 2 DOE Joint Genome Institute, Walnut Creek, California, USA; 3 Los Alamos National Laboratory, Bioscience Division, Los Alamos, New Mexico, USA; 4 Biological Data Management and Technology Center, Lawrence Berkeley National Laboratory, Berkeley, California, USA; 5 Department of Cell Biology and Biophysics, Faculty of Biology, University of Athens, Athens, Greece; 6 Centre for Bioinformatics - Department of Informatics - School of Natural & Mathematical Sciences, King's College London (KCL) - London, UK; 7 Present address: Computational Genomics Unit, Institute of Agrobiotechnology - Centre for Research & Technology Hellas - Thessaloniki - Greece

**Keywords:** Arthrobacter, dioxygenases, PAH biodegradation, phenanthrene degradation

## Abstract

*Arthrobacter phenanthrenivorans* is the type species of the genus, and is able to metabolize phenanthrene as a sole source of carbon and energy. *A. phenanthrenivorans* is an aerobic, non-motile, and Gram-positive bacterium, exhibiting a rod-coccus growth cycle which was originally isolated from a creosote polluted site in Epirus, Greece. Here we describe the features of this organism, together with the complete genome sequence, and annotation.

## Introduction

Strain Sphe3^T^ (=DSM 18606^T^ = LMG 23796^T^) is the type strain of *Arthrobacter phenanthrenivorans* [1]. It was isolated from Perivleptos, a creosote polluted site in Epirus, Greece (12 Km North of the city of Ioannina), where a wood preserving industry was operating for over 30 years [2]. Strain Sphe3^T^ is of particular interest because it is able to metabolize phenanthrene at concentrations of up to 400 mg/L as a sole source of carbon and energy, at rates faster than those reported for other *Arthrobacter* species [3-5]. It appears to internalize phenanthrene with two mechanisms: a passive diffusion when cells are grown on glucose, and an inducible active transport system, when cells are grown on phenanthrene as a sole carbon source [2]. Here we present a summary classification and a set of features for *A. phenanthrenivorans* strain Sphe3^T^, together with the description of the complete genome sequencing and annotation.

## Classification and features

**Figure 1:**
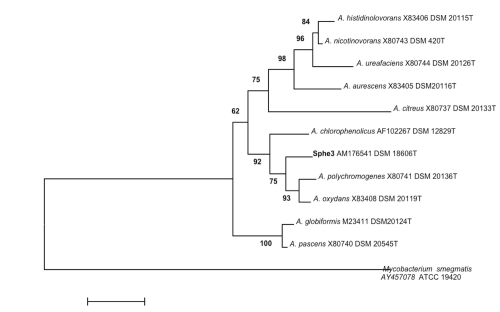
Phylogenetic tree highlighting the position of *A. phenanthrenivorans* strain Sphe3^T^ relative to the other type strains within the family. Numbers above branches are support values from 100 bootstrap replicates.

Figure 1 shows the phylogenetic neighborhood of *A. phenanthrenivorans* strain Sphe3^T^ in a 16S rRNA based tree.

**Figure 2:**
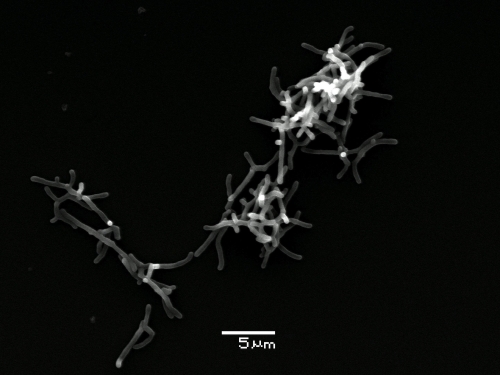
Scanning electron micrograph of *A. phenanthrenivorans* strain Sphe3^T^

Strain Sphe3^T^ is a Gram-positive, aerobic, non-motile bacterium exhibiting a rod-coccus cycle (Figure 2), with a cell size of approximately 1.0-1.5 x 2.5-4.0 μm. Colonies were slightly yellowish on Luria agar. The temperature range was 40-37^o^C with optimum growth at 30-37^o^C. The pH range was 6.5-8.5 with optimal growth at pH 7.0-7.5 (Table 1). Strain Sphe3^T^ was found to be sensitive to various antibiotics, the minimal inhibitory concentrations of which were estimated as follows: ampicillin 20 mgL^-1^, chloramphenicol 10 mgL^-1^, erythromycin 10 mgL^-1^, neomycin 20 mgL^-1^, rifampicin 10 mgL^-1^ and tetracycline 10 mgL^-1^.

**Table 1:** Classification and general features of *A. phenanthrenivorans* strain Sphe3^T^ according to the MIGS recommendations [6]

MIGS ID	Property	Term	Evidence code
	Current classification	Domain *Bacteria*	TAS [7]
		Phylum *Actinobacteria*	TAS [8]
		Class *Actinobacteria*	TAS [9]
		Subclass *Actinobacteridae*	TAS [9,10]
		Order *Actinomycetales*	TAS [9-12]
		Family *Micrococcaceae*	TAS [9-11,13]
		Genus *Arthrobacter*	TAS [1,11,14-17]
		Species *Arthrobacter phenanthrenivorans*	TAS [1]
		Type strain Sphe3	TAS [1]
	Gram stain	positive	TAS [1]
	Cell shape	irregular rods, coccoid	TAS [1]
	Motility	Non motile	TAS [1]
	Sporulation	nonsporulating	NAS
	Temperature range	mesophile	TAS [1]
	Optimum temperature	30°C	TAS [1]
	Salinity	normal	TAS [1]
MIGS-22	Oxygen requirement	aerobic	TAS [1]
	Carbon source	Phenanthrene, glucose, yeast extract	TAS [1,2]
	Energy source	Phenanthrene, glucose, yeast extract	TAS [1,2]
MIGS-6	Habitat	Soil	TAS [1,2]
MIGS-15	Biotic relationship	Free-living	NAS
MIGS-14	Pathogenicity	none	NAS
	Biosafety level	1	NAS
	Isolation	Creosote contaminated soil	TAS [1,2]
MIGS-4	Geographic location	Perivleptos, Epirus, Greece	TAS [1,2]
MIGS-5	Sample collection time	April 2000	TAS [1,2]
MIGS-4.1	Latitude	39.789	NAS
MIGS-4.2	Longitude	20.781	NAS
MIGS-4.3	Depth	10-20 cm	TAS [1,2]
MIGS-4.4	Altitude	500 meters	TAS [1,2]

Evidence codes - IDA: Inferred from Direct Assay (first time in publication); TAS: Traceable Author Statement (i.e., a direct report exists in the literature); NAS: Non-traceable Author Statement (i.e., not directly observed for the living, isolated sample, but based on a generally accepted property for the species, or anecdotal evidence). These evidence codes are from of the Gene Ontology project. If the evidence code is IDA, then the property was directly observed by one of the authors or an expert mentioned in the acknowledgements.

Amylase, catalase and nitrate reductase tests were positive, whereas arginine dihydrolase, gelatinase, lipase, lysine and ornithine decarboxylase, oxidase, urease, citrate assimilation and H_2_S production tests were negative. No acid was produced in the presence of glucose, lactose and sucrose.

### Chemotaxonomy

Menaquinones are the sole respiratory lipoquinones of *A. phenanthrenivorans* strain Sphe3^T^. Both MK-8 and MK-9(H_2_) are present in a ratio of 3.6:1, respectively. Major fatty acids are anteiso-C_15:0_ (36.2%), iso-C_16:0_ (15.7%), iso-C_15:0_ (14.3%), anteiso-C_17:0_ (12.0%), C_16:0_ (8.3%), iso-C_17:0_ (4.0%), C_16:1_ω7c (2.5%) and C_14:0_ (1.4%). The major phospholipids were diphospatidylglycerol (DPG), phosphatidylglycerol (PG) and phosphatidylethanolamine (PE), (63.8, 27.5 and 4.0% respectively).

## Genome sequencing and annotation

### Genome project history

This organism was selected for sequencing on the basis of its biodegradation capabilities, i.e. metabolizes phenanthrene as a sole source of carbon and energy. The genome project is deposited in the Genome OnLine Database [18] and the complete genome sequence is deposited in GenBank. Sequencing, finishing and annotation were performed by the DOE Joint Genome Institute (JGI). A summary of the project information is shown in Table 2.

**Table 2:** Genome sequencing project information

**MIGS ID**	**Property**	**Term**
MIGS-31	Finishing quality	Finished
MIGS-28	Libraries used	Three genomic libraries: 6kb (pMCL200) and fosmids (pcc1Fos) Sanger libraries and one 454 pyrosequence standard library
MIGS-29	Sequencing platforms	ABI 3730. 454 GS FLX
MIGS-31.2	Sequencing coverage	9.33× Sanger, 17.45× pyrosequence
MIGS-30	Assemblers	Newbler version 1.1.02.15, Arachne
MIGS-32	Gene calling method	Prodigal, GenePRIMP
	INSDC ID	CP002379
	Genbank Date of Release	February 16, 2011
	GOLD ID	Gc01621
	NCBI project ID	38025
	Database: IMG-GEBA	2503538005
MIGS-13	Source material identifier	DSM 12885
	Project relevance	Tree of Life, GEBA

### Growth conditions and DNA isolation

*A. phenanthrenivorans* Sphe3^T^, DSM 18606^T^ was grown aerobically at 30°C on MM M9 containing 0.02% (w/v) phenanthrene. DNA was isolated according to the standard JGI (CA, USA) protocol for Bacterial genomic DNA isolation using CTAB.

### Genome sequencing and assembly

The genome of *Arthrobacter phenanthrenivorans* type strain (Sphe3)was sequenced using a combination of Sanger and 454 sequencing platforms. All general aspects of library construction and sequencing can be found at the JGI website [19]. Pyrosequencing reads were assembled using the Newbler assembler version 1.1.02.15 (Roche). Large Newbler contigs were broken into 4,967 overlapping fragments of 1,000 bp and entered into assembly as pseudo-reads. The sequences were assigned quality scores based on Newbler consensus q-scores with modifications to account for overlap redundancy and to adjust inflated q-scores. A hybrid 454/Sanger assembly was made using the Arachne assembler [20]. Possible mis-assemblies were corrected and gaps between contigs were closed by by editing in Consed, by custom primer walks from sub-clones or PCR products. A total of 822 Sanger finishing reads were produced to close gaps, to resolve repetitive regions, and to raise the quality of the finished sequence. The error rate of the completed genome sequence is less than 1 in 100,000. Together, the combination of the Sanger and 454 sequencing platforms provided 26.78 x coverage of the genome. The final assembly contains 44,113 Sanger reads and 599,557 pyrosequencing reads.

### Genome annotation

Genes were identified using Prodigal [21] as part of the Oak Ridge National Laboratory genome annotation pipeline, followed by a round of manual curation using the JGI GenePRIMP pipeline [22]. The predicted CDSs were translated and used to search the National Center for Biotechnology Information (NCBI) nonredundant database, UniProt, TIGR-Fam, Pfam, PRIAM, KEGG, COG, and InterPro databases. Additional gene prediction analysis and functional annotation were performed within the Integrated Microbial Genomes - Expert Review (IMG-ER) platform [23].

## Genome properties

**Figure 3:**
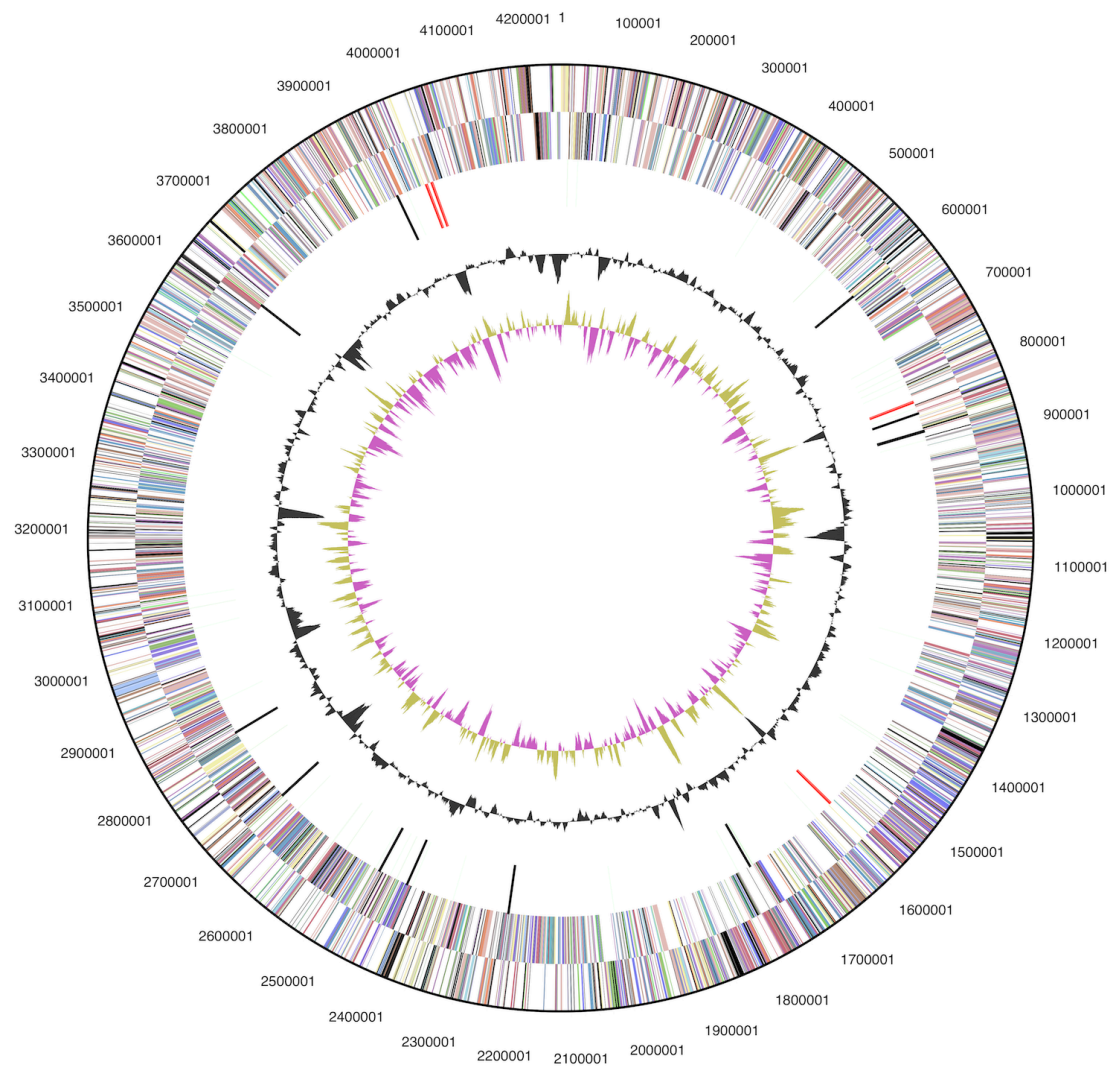
Graphical circular map of the chromosome, not drawn to scale with plasmids. From outside to the center: Genes on forward strand (color by COG categories), Genes on reverse strand (color by COG categories), RNA genes (tRNAs green, rRNAs red, other RNAs black), GC content, GC skew.

**Figure 4:**
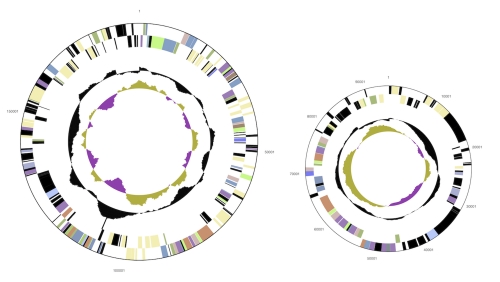
The two plasmids, not drawn to scale with chromosome. From outside to the center: Genes on forward strand (color by COG categories), Genes on reverse strand (color by COG categories), RNA genes (tRNAs green, rRNAs red, other RNAs black), GC content, GC skew.

The genome consists of a 4,250,414 bp long chromosome with a GC content of 66% and two plasmids both with 62% GC content, the larger being 190,450 bp long and the smaller 94,456 bp (Table 3, Figure 3 and Figure 4). Of the 4,288 genes predicted, 4,212 were protein-coding genes, and 76 RNAs; 77 pseudogenes were also identified. The majority of the protein-coding genes (73.8%) were assigned with a putative function while the remaining ones were annotated as hypothetical proteins. The distribution of genes into COGs functional categories is presented in Table 4.

**Table 3:** Genome Statistics

**Attribute**	Value	% of Total
Genome size (bp)	4,535,320	100.00%
DNA Coding region (bp)	4,033,112	88.93%
DNA G+C content (bp)	2,964,596	65.37%
Number of replicons	1	
Extrachromosomal elements	2	
Total genes	4,288	100.00%
RNA genes	76	1.77%
rRNA operons	4	
Protein-coding genes	4,212	98.23%
Pseudo genes	77	1.80%
Genes with function prediction	3,167	73.86%
Genes in paralog clusters	930	21.69%
Genes assigned to COGs	3,075	71.71%
Genes assigned Pfam domains	3,277	76.42%
Genes with signal peptides	978	22.81%
Genes with transmembrane helices	999	23.30%
CRISPR repeats	0	

**Table 4:** Number of genes associated with the general COG functional categories

**Code**	**value**	**%age**	**Description**
J	153	4.5	Translation, ribosomal structure and biogenesis
A	1	0.0	RNA processing and modification
K	308	9.0	Transcription
L	239	7.0	Replication, recombination and repair
B	1	0.0	Chromatin structure and dynamics
D	29	0.8	Cell cycle control, cell division, chromosome partitioning
Y	0	0.0	Nuclear structure
V	45	1.3	Defense mechanisms
T	135	3.9	Signal transduction mechanisms
M	142	4.1	Cell wall/membrane/envelope biogenesis
N	2	0.0	Cell motility
Z	0	0.0	Cytoskeleton
W	0	0.0	Extracellular structures
U	45	1.3	Intracellular trafficking and secretion, and vesicular transport
O	100	2.9	Posttranslational modification, protein turnover, chaperones
C	205	6.0	Energy production and conversion
G	396	11.6	Carbohydrate transport and metabolism
E	329	9.6	Amino acid transport and metabolism
F	87	2.5	Nucleotide transport and metabolism
H	141	4.2	Coenzyme transport and metabolism
I	134	3.9	Lipid transport and metabolism
P	167	4.9	Inorganic ion transport and metabolism
Q	95	2.8	Secondary metabolites biosynthesis, transport and catabolism
R	430	12.6	General function prediction only
S	238	6. 9	Function unknown
-	1,213	28.3	Not in COGs
